# A comparison of different DNA extraction methods and molecular techniques for the detection and identification of foodborne pathogens

**DOI:** 10.3934/microbiol.2021019

**Published:** 2021-09-09

**Authors:** Spyridon Andreas Papatheodorou, Panagiotis Halvatsiotis, Dimitra Houhoula

**Affiliations:** 1 Department of Food Science & Technology, School of Food Sciences, University of West Attica; 2 2nd Propaedeutic Department of Internal Medicine, Medical School, National and Kapodistrian University of Athens, “ATTIKON” University Hospital, Chaidari Greece

**Keywords:** DNA extraction methods, *Salmonella enterica* serovar Typhimurium, *Listeria monocytogenes*, PCR, LAMP, Limit of detection

## Abstract

Foodborne infections continue to plague Europe. Food safety monitoring is in crisis as the existing techniques for detecting pathogens do not keep up with the global rising of food production and consumption. Thus, the development of innovative techniques for detecting and identifying pathogenic bacteria has become critical. The aim of the present study was firstly to develop an innovative simple and low cost method of extracting bacterial DNA from contaminated food and water samples with *Salmonella enteric(a)* subsp. *enteric(a)* serovar Typhimurium and *Listeria monocytogenes* and its comparison with two commercial DNA extraction kits (Qiagen, Macherey-Nagel). Finally, pathogens' detection using two molecular techniques (PCR-electrophoresis, LAMP), in order to evaluate the best combination of DNA extraction and identification based on their sensitivity, cost, rapidity and simplicity. Considering the above criteria, among them, best was proved an in-house bacterial DNA extraction method, based on the chloroform-isoamyl alcohol protocol, with certain modifications. This technique showed statistically similar results in terms of sensitivity, compared to the commercial kits, while at the same time maintained high rapidity and much lower cost. Lastly, between the molecular techniques, LAMP was found more promising considering its simplicity, high rapidity and sensitivity. Conclusively, the in-house DNA extraction method along with the LAMP technique, was proven to be the best among the presented combinations.

## 1. Introduction

Foodborne diseases continue to pose a major threat to both the public health and the economy in Europe [1]. Even though the precautionary measures are increasingly tightened [2], the number of outbreaks keep rising [3]. According to the European Food Safety Authority, amongst all foodborne bacterial illnesses, Salmonellosis and Listeriosis were responsible for the most reported deaths in the European Union in 2018 [4]. Thus, pathogenic bacteria such as *Salmonella spp*. and *Listeria monocytogenes* are a major hazard and their detection should be considered critical [5]–[8].

The conventional techniques for the detection of bacterial pathogens in food, although being trustworthy, require days to give results [9]. So, as the global food production increases, the conventional techniques do not seem to keep up. Thus, revolutionary, low cost and short integration times are considered essential for the identification of bacteria and their resistance genes. As a result, DNA-based techniques have been developed the past years in order to fill the existing gaps [10],[11]. Molecular techniques have been used for the detection of various foodborne pathogens due to their capability of analyzing many samples at once, their rapidity and their high sensitivity [9],[11],[12].

On the other hand, they also present certain disadvantages. Most molecular techniques require expensive equipment and trained personnel [13]. Moreover, the DNA isolation and extraction is still very costly, making the DNA-based methods inappropriate for fields of mass production. Furthermore, complexed matrices such as food may affect the result of most molecular techniques [14]. However, certain methods such as the Loop Mediated Isothermal Amplification (LAMP) are more tolerant to inhibitors [15]. This particular technique has a higher sensitivity than Polymerase Chain Reaction (PCR) since it requires more primers [16]. In addition, it is much simpler and operates in isothermal conditions (65 °C) for 30–60 min in total. This means that it can be conducted rapidly without the use of expensive equipment (e.g. thermal cycler) [17].

The scope of the present study was to develop an inexpensive, rapid and valid method of extracting bacterial DNA that could replace the commercial DNA isolation kits. This, combined with the colorimetric LAMP, a molecular technique known for its sensitivity and rapidity, could contribute greatly in minimizing each samples' cost and will be most promising to provide very fast, simple and cheap results without the need of expensive instrumentation. This will consist of an integrated, low-cost, platform, exploiting a universal assay strategy based on molecular diagnostics, which will provide a rapid result with a user-friendly and affordable procedure, aimed at large-scale applications in decentralized laboratories.

## 2. Materials and methods

### 2.1. Water and food samples contamination

For the purpose of the present study, water and food samples (milk and chicken) were spiked with *S*. Typhimurium (NCTC 12023) and *L. monocytogenes* (NCTC 10357). The bacterial suspensions were prepared in Tryptone Soy Broth (TSB) to a 0.5 McFarland standard turbidity equivalent (approx. 1.5 × 10^8^ cfu/mL) and tenfold serial dilutions were prepared in 10 mL final volume, to a final concentration of 1.5 × 10^1^ cfu/mL in the above matrices. Each serial dilution was later analyzed, in order to evaluate the analytical sensitivity.

### 2.2. Food collection

During the study period (February to September 2020) a total of 60 food specimens (more specifically 15 chicken and 45 milk products) were collected from local markets and were analyzed for detection of *S*. Typhimurium and *L. monocytogenes* respectively.

### 2.3. Microbiological tests

Each sample was submitted to tenfold serial dilutions and was microbiologically tested in order to evaluate the analytical sensitivities of the above methods. From each sample, 25 grams were weighted in sterile bags and 225 mL of Peptone buffered Water (BPW) were added and homogenized in a Stomacher (Laboratory Blender Stomacher 400, Seward Medical, England) for 30 seconds. For the identification of *L. monocytogenes* 0.1 mL was transferred to 9.9 mL BPW and then 0.1 mL was transferred to selective CHROMagar™ LISTERIA (Bioprepare Microbiology, Athens, Greece) in order to quantify the cells. For the identification of *S*. Typhimurium, following 24 hours of pre-enrichment in BPW, 0.1 mL of sample was transferred to selective CHROMagar™ SALMONELLA PLUS (Bioprepare Microbiology, Athens, Greece)

### 2.4. Genomic DNA extraction methods

After spiking of pathogenic bacteria in water and food matrices four different bacterial DNA extraction methods were used and evaluated in each spiked sample.

Methods 1 and 2 were applied according to the manufacturer's protocol of two commercial kits, DNeasy blood and tissue kit (Qiagen Ltd., West Sussex, United Kingdom) (After many studies conducted in our laboratory, we have noticed from collected unpublished data that blood and tissue kit presents similar performance for the extraction of DNA from Food samples and at the same time it is less expensive in comparison to other commercial kits that specialize in DNA extraction from food samples) and NucleoSpin® Food (Macherey-Nagel, Duren, Germany).

Method 3 relied on cell wall disruption by boiling of each sample for 10 min and ultrasonic bathing (Elma TI-H 10, Germany) for additional 10min with a power of 200 w. Eventually, each sample was centrifuged at 11.000 rpm for 2 min and the aqueous phase was collected.

Method 4 was an in-house extraction method, based on the standard phenol-chloroform-isoamyl alcohol protocol [18] with certain modifications. Briefly, 1 mL of each water and the suspension of food was boiled in a water bath for 10 min and then put into ultrasonic bath for additional 10 min in order to cause cell membrane lysis without the use of a chaotropic agent (e.g., phenol). The mixture was centrifuged (SL 16R, Thermo Scientific, Germany) at 11.000 rpm for 1 min and the aqueous phase was collected. An equal volume of chloroform-isoamyl alcohol solution (0.96:0.04) was added and centrifuged at 11.000 rpm for 1 min. The aqueous (upper) phase was collected and an equal volume of chloroform was added and put for another centrifugation at 11.000 rpm for 1 min. After collection of the aqueous phase, 50 µL of sodium acetate 3 M (pH = 5.2) was also added to the solution. An equal volume of absolute ethanol (at room temperature)was added to the mixture in order to precipitate the DNA. The mixture was centrifuged at 11.000 rpm for 1 min and the liquid phase was removed. Additional 250 µL of absolute ethanol was added and left under freezing conditions (−20 °C) for 30 min. Afterwards, the mixture was put for centrifugation at 11.000 rpm for 2 min. The ethanol was also removed and the samples were heated at 60 °C in a heater block (HB-M501, Italy) in order for the remaining ethanol to evaporate. Finally, the DNA pellet was resuspended in 100 µL elution buffer (10 mM Tris-HCl, pH = 8.0, 1 mM EDTA) and collected in 1.5 µL Eppendorf microtubes.

### 2.5. PCR-Electrophoresis

Following the bacterial DNA extraction for each sample, the next step consisted of PCR amplification. PCR was performed in 50µL final volume solution for each sample consisting of 25 µL OneTaq® Hot Start Quick-Load 2X Master Mix with Standard Buffer (NEB), 0.4 µM primers (for *S*. Typhimurium/*L. monocytogenes*) as described by Anju, Latha, Sunil et al., 2014 [20] and Khalil 2017 [21] respectively, 5 µL of the target bacterial DNA and UltraPure™ DNase/RNase-Free Water (Invitrogen) up to a final volume of 50 µL.

The procedure was carried out on a Veriti® 96 Well Therman Cycler (Applied Biosystems®). The amplification conditions were adjusted as follows: Initially, a denaturation step at 95 °C for 10 min, followed by 40 cycles of: 60 sec denaturation at 95 °C and annealing for 60 sec at 58 °C for *S*. Typhimurium or 52 °C for L. monocytogenes, elongation at 72 °C for 60 sec and final elongation at 72 °C for another 7 min. The PCR products were separated by electrophoresis in 1.5% agarose gel, stained with ethidium bromide (0.5 µg/mL) and documented under UV illumination using MiniBIS Pro device.

### 2.6. Loop Mediated Isothermal Amplification (LAMP)

A Loop Mediated Isothermal Amplification, as described by Tanner N.A., Zhang Y. & Evans, T.C. 2015 [19] was transacted to compare the sensitivity of each molecular method. The LAMP was performed in 25 µL final volume solution for each sample, consisting of 12.5 µL WarmStart® Colorimetric LAMP 2X Master Mix (NEB), 0.8 *µ*M each of FIP and BIP primers, 0.1 µM each of F3 and B3 primers and 0.4 µM each of LF and LB primers for *S*. Typhimurium (Srisawa & Panbangred, 2015) and *L. monocytogenes* [23] as shown in table 1, 5 µL target bacterial DNA and UltraPure™ DNase/RNase-Free Water (Invitrogen) up to 25 µL. The amplification was carried out in a conventional oven set at 65 °C for 45 min for the samples contaminated with *S*. Typhimurium and 60 min for the samples contaminated with *L. monocytogenes* respectively. The results were evaluated based on the color change without use of any additional instrumentation.

### 2.7. Statistical analysis

The experiments were conducted five times and statistical analysis of the data was performed using the software Statistica 6.0 (StatSoft, Tulsa, OK, USA). Data measurements were subjected to variance analysis for interaction of four different effects (extraction method, molecular technique, matrix and pathogen) in combination with two molecular techniques (PCR-electrophoresis, LAMP) in the final limit of detection. The variables were also subjected to Duncan's multiple-range test.

**Table 1.** Nucleotide sequences of LAMP primers used for the detection of *S. enterica* serovar Typhimurium and *L. monocytogenes* [22],[23].

Target gene	Primer	Sequence (5′-3′)	Length
*S*. Typhimurium			
*Stn*	F3	5′ ACCAGATTCAGGGAGTGAGT 3′	20
	B3	5′ CGCGCACGAAATTCGTAAC 3′	19
	FIP	5′ ACCGGGTGGTAAGCGAATTGCGAGGTTAACCGTCTGGAGC 3′	40
	BIP	5′ TCGGCCTCTTTGGCCATCACTGGCGAAATACTTTGCCGAG 3′	40
	LF	5′ TGGTAAAGCCCGCGCATCTG 3′	20
	LB	5′ GCGCCAGTTCATGCGACTCG 3′	20

*L. monocytogenes*			
*hlyA*	F3	5′ TTGCGCAACAAACTGAAGC 3′	19
	B3	5′ GCTTTTACGAGAGCACCTGG 3′	20
	FIP	5′ CGTGTTTCTTTTCGATTGGCGTCTTTTTTTCATCCATGGCACCACC 3′	46
	BIP	5′ CCACGGAGATGCAGTGACAAATGTTTTGGATTTCTTCTTTTTCTCCACAAC 3′	51
	LF	5′ TAGGACTTGCAG GCGGAGATG 3′	21
	LB	5′ GCCAAGAAAAGGTTACAAAGATGG 3′	24

### 2.8. Food specimens

After completion and comparison of the DNA extraction methods (methods one to four, section 2.4) and the molecular techniques used (PCR-electrophoresis and LAMP), all the commercial food specimens were subjected to DNA extraction (using method 4, section 2.4) and identification (with both molecular techniques: PCR-electrophoresis and LAMP).

## 3. Results

### 3.1. Microbiological tests

Commercial food samples were collected from local supermarkets and were tested for *S*. Typhimurium and *L. monocytogenes* presence (section 2.2). From the 60 food samples collected from the local Markets, all were negative in *S*. Typhimurium by microbial culture. Likewise for the *L. monocytogenes*, from the 15 tested chicken samples, 3 were positive while from the rest 45 milk samples, 3 were also positive.

### 3.2. PCR assay

The extracted bacterial DNA was carried out using OneTaq® Hot Start Quick-Load 2X Master Mix with Standard Buffer and was subjected to PCR-Electrophoresis tests. The limit of detection of each extraction method in every matrix is presented at Table 2 and the electrophoresis is presented at Figure 1(a) and 1(b).

**Table 2.** Limit of Detection and Sensitivity of foodborne pathogens (*1.5 cfu/mL) in spiked food matrices, according to the studied extraction methods and molecular techniques.

			PCR	LAMP
chicken	*L. monocytogenes*	iv	10^3Aab^	10^2Aab^
		iii	10^4Bab^	10^4Bab^
		ii	10^2Aab^	10^2Aab^
		i	10^2Aab^	10^1Aab^
	*S. enterica* serovar Typhimurium	iv	10^3Aab^	10^2Aab^
		iii	10^4Bab^	10^4Bab^
		ii	10^2Aab^	10^1Aab^
		i	10^2Aab^	10^1Aab^
milk	*L. monocytogenes*	iv	10^4Ab^	10^3Ab^
		iii	10^5Bb^	10^4Bb^
		ii	10^2Ab^	10^2Ab^
		i	10^2Ab^	10^2Ab^
	*S. enterica* serovar Typhimurium	iv	10^3Ab^	10^2Ab^
		iii	10^5Bb^	10^4Bb^
		ii	10^2Ab^	10^2Ab^
		i	10^2Ab^	10^2Ab^
water	*L. monocytogenes*	iv	10^2Aa^	10^1Aa^
		iii	10^2Ba^	10^2Ba^
		ii	10^2Aa^	10^1Aa^
		i	10^2Aa^	10^1Aa^
	*S. enterica* serovar Typhimurium	iv	10^2Aa^	10^1Aa^
		iii	10^2Ba^	10^2Ba^
		ii	10^2Aa^	10^1Aa^
		i	10^2Aa^	10^1Aa^

i: DNeasy blood and tissue kit (Qiagen Ltd., West Sussex, United Kingdom)

ii: NucleoSpin® Food (Macherey-Nagel, Duren, Germany)

iii: Boil – ultrasonication – centrifuge (method 3, chapter 2.4)

iv: In-house extraction method based on the standard phenol-chloroform-isoamyl alcohol protocol (method 4, chapter 2.4) with method 4, chapter 2.4.

**Figure 1a.**
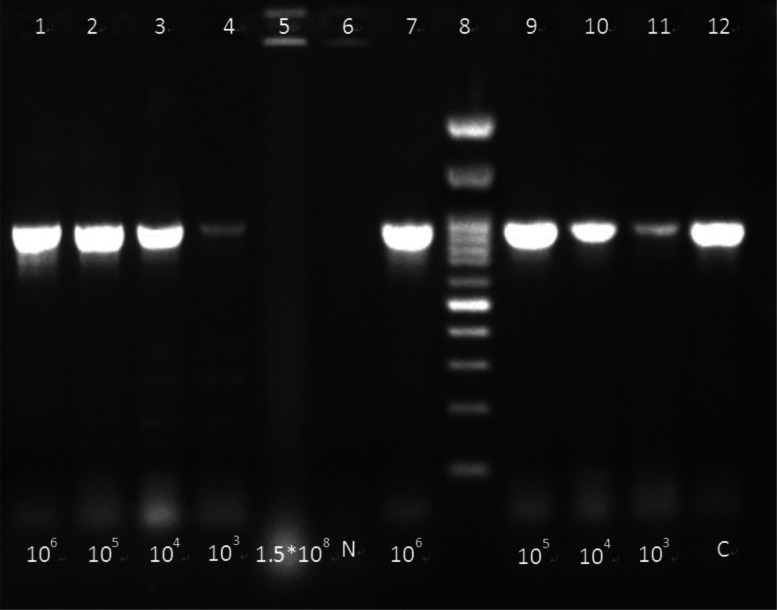
Electrophoresis of food samples spiked with *S. enterica* serovar Typhimurium (*1.5 cfu/mL) after bacterial DNA extraction with method 4, chapter 2.4.

**Figure 1b.**
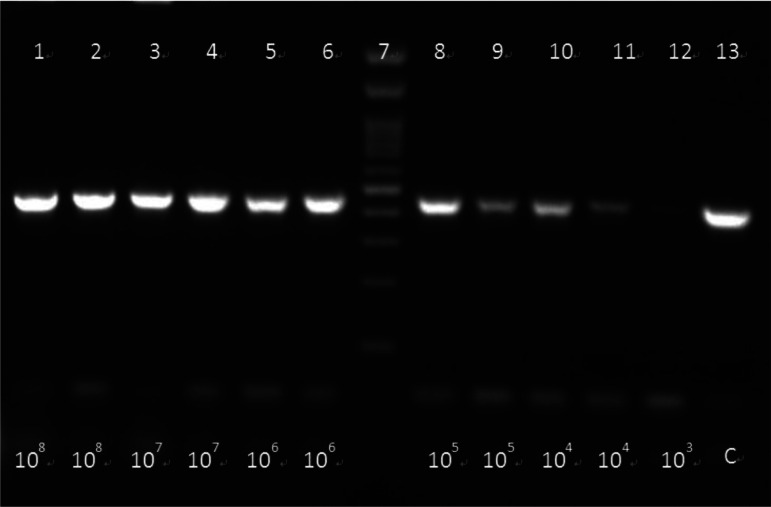
Electrophoresis of food samples spiked with *L. monocytogenes* (*1.5 cfu/mL) after bacterial DNA extraction with method 4, chapter 2.4.

**Figure 1c.**
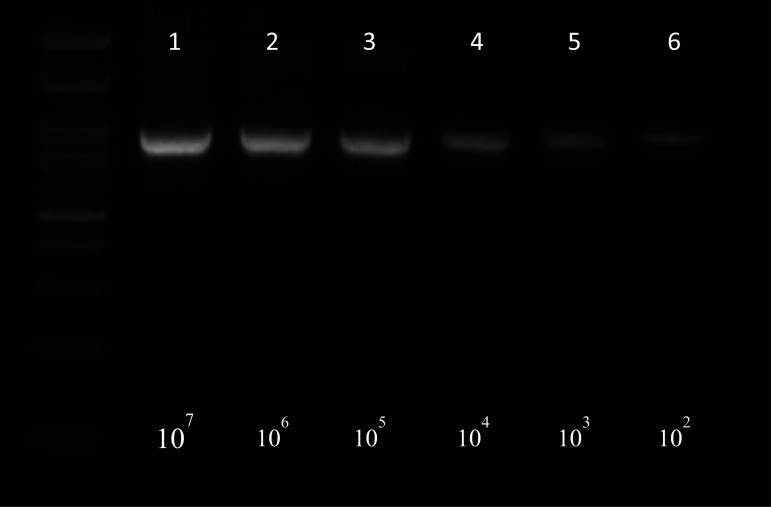
Electrophoresis of water samples spiked with *S*. Typhimurium (*1.5 cfu/mL) after bacterial DNA extraction with method 4, chapter 2.4.

**Figure 1d.**
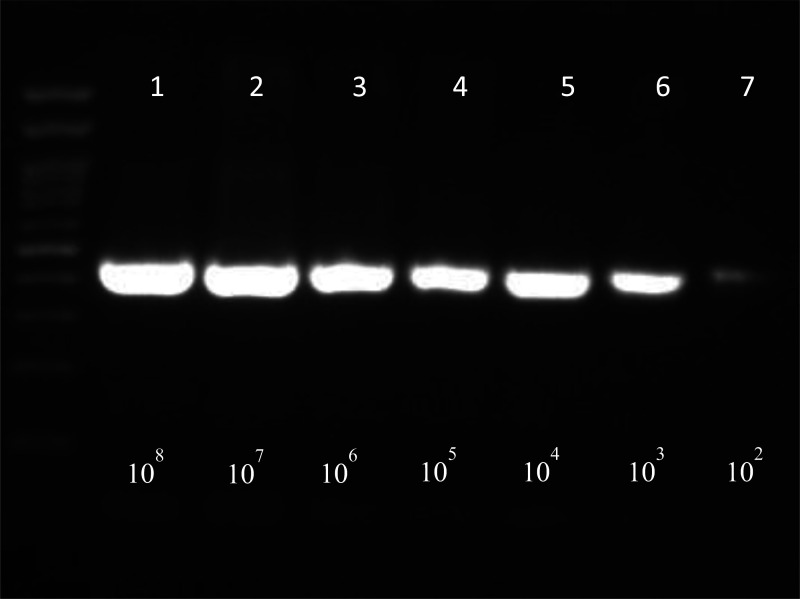
Electrophoresis of water samples spiked with *L. monocytogenes* (*1.5 cfu/mL) after bacterial DNA extraction with method 4, chapter 2.4.

Figure 1(a) shows detection of *S*. Typhimurium spiked in whole milk and chicken breast samples, starting from an initial concentration of 1.5*10^6^ cfu/mL followed by serial tenfold dilutions until a final concentration of 1.5*10^3^ cfu/mL. The samples shown are as follows: 1: *S*. Typhimurium in milk (1.5*10^6^ cfu/mL). 2: *S*. Typhimuriumin milk (1.5*10^5^ cfu/mL). 3: *S*. Typhimurium in milk (1.5*10^4^cfu/mL). 4: *S*. Typhimuriumin milk (1.5*10^3^ cfu/mL) 5: *S*. Typhimurium in milk (1.5·10^8^ cfu/mL). 6: Negative sample. 7: *S*. Typhimurium in chicken (1.5*10^6^ cfu/mL). 8: 100 bp DNA Ladder (Invitrogen, Thermo Fischer Scientific). 9: *S*. Typhimurium in chicken (1.5*10^5^ cfu/mL). 10: *S*. Typhimurium in chicken (1.5*10^4^ cfu/mL). 11: *S*. Typhimurium in chicken (1.5*10^3^ cfu/mL). 12: control sample. While the rest were detected successfully at 915 bp, the sample in the fifth well (1.5*10^8^ cfu/mL) was found negative. This probably happened because of inhibitors that could possibly be present in samples with large amounts of DNA.

Respectively, Figure 1(b) shows detection of *L. monocytogenes* spiked in the same samples as Figure 1(a) (1: *L. monocytogenes* in chicken (1.5*10^8^ cfu/mL). 2: *L. monocytogenes* in milk (1.5*10^8^ cfu/mL). 3: *L. monocytogenes* in chicken (1.5*10^7^ cfu/mL). 4: *L. monocytogenes* in milk (1.5*10^7^ cfu/mL) 5: *L. monocytogenes* in chicken (1.5*10^6^ cfu/mL). 6: *L. monocytogenes* in milk (1.5*10^6^ cfu/mL). 7: 100bp DNA Ladder (Invitrogen, Thermo Fischer Scientific).8: *L. monocytogenes* in chicken (1.5*10^5^ cfu/mL). 9: *L. monocytogenes* in milk (1.5*10^5^ cfu/mL). 10: *L. monocytogenes* in chicken (1.5*10^4^ cfu/mL). 11: *L. monocytogenes* in milk (1.5*10^4^ cfu/mL). 12: *L. monocytogenes* in chicken (1.5*10^3^ cfu/mL) 13: control sample. The extraction was carried out according to method 4, chapter 2.4). The detection was performed starting from initial concentrations of 1.5*10^8^ cfu/mL. Subsequently, serial tenfold dilutions were also performed and detected up to a final concentration of 1.5*10^3^ cfu/mL. All samples were found positive at 454 bp.

Among all the tested commercial samples, 3 chicken and 3 milk samples were positive for *L. monocytogenes* with the PCR assay. The positive results were also the same as those with the microbiological analyzes.

Figure 1(c) shows detection of *S*. Typhimurium spiked in water samples starting from an initial concentration 1.5*10^7^ cfu/mL followed by serial tenfold dilutions until a final concentration of 1.5*10^2^ cfu/mL. All samples were found positive at 915 bp.

Figure 1(d) shows detection of *L. monocytogenes* spiked in water samples starting from an initial concentration 1.5*10^8^ cfu/mL followed by serial tenfold dilutions until a final concentration of 1.5*10^2^ cfu/mL. All samples were found positive at 454 bp.

### 3.3. LAMP assay

The LAMP assay was carried out using the WarmStart® Colorimetric LAMP 2X Master Mix and the samples were incubated in a conventional oven. The optimal time-temperature ratio was 65 °C for 45min for the samples spiked with *S*. Typhimurium and 65 °C for 60 min for the samples spiked with *L. monocytogenes*. The final volume of each reaction was 25 µL. The limit of detection of each extraction method in every matrix is presented at Table 2.

**Figure 2a.**
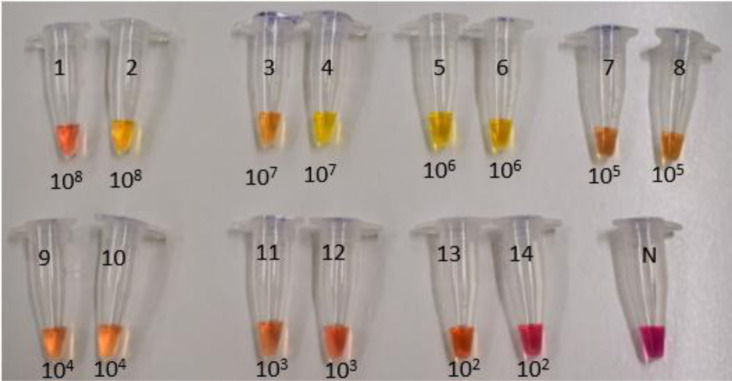
LAMP in food samples spiked with *S*. Typhimurium and *L. monocytogenes* (*1.5 cfu/mL) after bacterial DNA extraction with method 4, chapter 2.2.

**Figure 2b.**
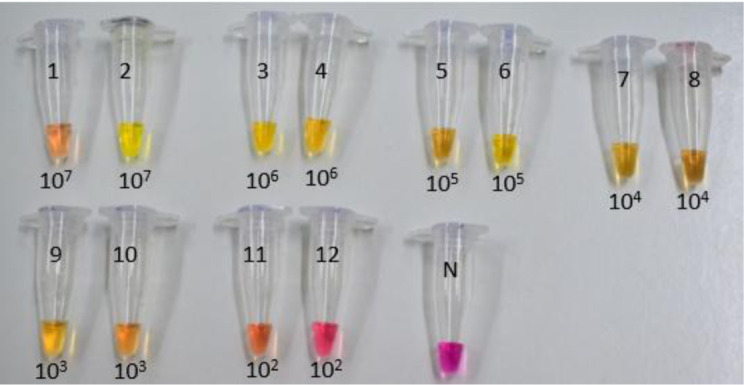
LAMP in food samples spiked with *S*. Typhimurium and *L. monocytogenes* (*1.5 cfu/mL) after bacterial DNA extraction with method 4, chapter 2.2.

Figures 2(a) and 2(b) show the identification of food samples (milk, chicken) by the LAMP assay, spiked with *S. S*. Typhimurium and *L. monocytogenes:* Figure 2(a): 1: *S*. Typhimuriumin milk (1.5*10^8^ cfu/mL). 2: *L. monocytogenes* in milk (1.5*10^8^ cfu/mL). 3: *S*. Typhimurium in milk (1.5*10^7^ cfu/mL). 4: *L. monocytogenes* in milk (1.5*10^7^ cfu/mL). 5: *S*. Typhimuriumin milk (1.5*10^6^ cfu/mL). 6: *L. monocytogenes* in milk (1.5*10^6^ cfu/mL). 7: *S*. Typhimuriumin milk (1.5*10^5^ cfu/mL). 8: *L. monocytogenes* in milk (1.5*10^5^ cfu/mL). 9: *S*. Typhimuriumin milk (1.5*10^4^ cfu/mL). 10: *L. monocytogenes* in milk (1.5*10^4^ cfu/mL). 11: *S*. Typhimuriumin milk (1.5*10^3^ cfu/mL). 12: *L. monocytogenes* in milk (1.5*10^3^ cfu/mL). 13: *S*. Typhimuriumin milk (1.5*10^2^ cfu/mL). 14: *L. monocytogenes* in milk (1.5*10^2^ cfu/mL). 15: Negative Sample. Fig 2(b): 1: *S. S*. Typhimurium in chicken (1.5*10^7^ cfu/mL). 2: *L. monocytogenes* in chicken (1.5*10^7^ cfu/mL). 3: *S*. Typhimurium in chicken (1.5*10^6^ cfu/mL). 4: *L. monocytogenes* in chicken (1.5*10^6^ cfu/mL). 5: *S*. Typhimurium in chicken (1.5*10^5^ cfu/mL). 6: *L. monocytogenes* in chicken (1.5*10^5^ cfu/mL). 7: *S*. Typhimurium in chicken (1.5*10^4^ cfu/mL). 8: *L. monocytogenes* in chicken (1.5*10^4^ cfu/mL). 9: *S*. Typhimurium in chicken (1.5*10^3^ cfu/mL). 10: *L. monocytogenes* in chicken (1.5*10^3^ cfu/mL). 11: *S*. Typhimurium in chicken (1.5*10^2^ cfu/mL). 12: *L. monocytogenes* in chicken (1.5*10^2^ cfu/mL). 13: Negative Sample.

From the above figures, it can be seen that both food samples present the same LOD and the detection of *S*. Typhimurium has a slightly higher sensitivity than that of the *L. monocytogenes* with detection limits of 1.5*10^2^ cfu/mL and 1.5*10^3^ cfu/mL respectively. The possible reason for this small (but not statistically important) difference is that *S*. Typhimurium is gram negative while *L. monocytogenes* is gram positive bacteria. Gram positive bacteria have much thicker cell wall (20–40 nanometers thick) and thus are more durable to cell wall disruption procedures.

Positive samples are distinguished from negatives as the color of the pH indicator dyes changes from purple to yellow. This color change occurs due to the production of protons during the amplification of Bst DNA polymerase, in combination with the low concentration of buffer in the colorimetric LAMP mixture (Taner et al. 2015).

**Figure 3.**
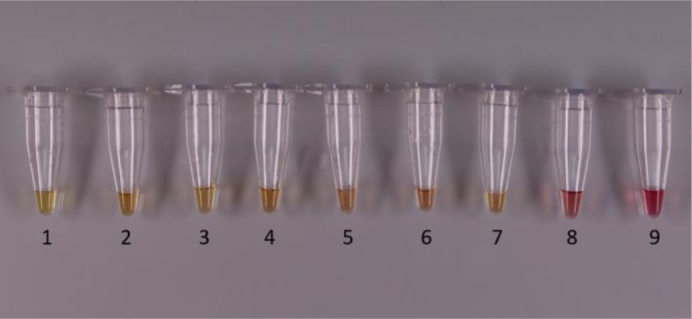
LAMP in commercial chicken and milk samples after bacterial DNA extraction with method 4, chapter 2.2.1–4: Commercial chicken samples tested for detection of L. monocytogenes. 4–6: Commercial milk samples tested for detection of L. monocytogenes. 7: Sample spiked with *L. monocytogenes* as control. 8: Negative sample (spiked with *S*. Typhimurium). 9: Negative sample (water).

Figure 3 shows detection of *L. monocytogenes* in commercial milk and chicken samples. From the above figure, all food samples 1–6, were positive for *L. monocytogenes*. Furthermore, the control sample (7) was also positive for *L. monocytogenes*, while the samples 8 and 9 were found negative as expected. The detection and showed the same results with the PCR-electrophoresis method and microbiological analysis, giving also 100% specificity.

### 3.4. Repeatability and reproducibility of PCR and LAMP assays

The specificity of the primer pair was confirmed by PCR amplification Extracted DNA from a 24 h culture of *Escherichia coli* ATCC 25922, and a clinical *Yersinia enterocolytica* strain (which were designated specificity controls), was used for specificity confirmation. Extraction was additionally performed using an equivalent volume of 10mM PBS solution (which was designated negative control).

For the evaluation of the repeatability and reproducibility of each extraction method, five samples at concentrations from 1.5 × 10^6^ cfu/mL to 1.5 × 10^2^ cfu/mL (with tenfold serial dilutions) were randomly chosen and amplified in triplicate with LAMP and PCR assays. All samples showed 100% repeatability and reproducibility.

### 3.5. Statistical processing

Based on the analysis of the bacterial strains used, the PCR-electrophoresis and LAMP assays were 100% specific, since no negative results were found positive. From all the studied variables (extraction method, molecular technique, matrix and bacterial pathogen), the extraction method significantly affected the limit of detection of the samples (P < 0.05). After submitting the results to Duncan's new multiple range test, it is observed that, with respect to variable 1 (bacterial DNA extraction method), the methods i, ii and iv (Table 2), do not present significant differences (Category A), while method iii (Table 2) gives significantly higher limit of detection (p < 0.05) than the rest (category B). In addition, for variable 3 (food matrix), the water (category a) matrix presents a significantly lower detection limit (p < 0,05) than the whole milk matrix (category b). However the chicken breast matrix does not present any statistically significant differences compared to any of the other 2 matrices. (Category ab).

### 3.6. Cost and time consumption of each DNA extraction method

**Table 3.** Time consumption and cost of each DNA extraction method.

Extraction method	Time (min)	Cost per sample (€)
1 Qiagen	75	3.92
2 MN	75	2.7
3 boil – us	25	-
4 in-house	80	<1

## 4. Discussion

In Europe, *S. enterica* serovar Typhimurium and *Listeria monocytogenes* are emerging as drivers of most of the severe foodborne pathogens.The classical techniques for foodborne pathogens detection require a great amount of time to conduct while the molecular techniques are much more expensive and thus are not preferred by food companies. In this paper we report on the detection of *S*. Typhimurium *L. monocytogenes* in food samples by LAMP and PCR-electrophoresis by using four different extraction DNA methods. The commercial QIAamp and Nucleospin DNA purification procedure comprise three steps using spin columns with silica-gel membrane. The first step is that with the lysate buffering conditions DNA absorbed into the column. In continuous the DNA bound to the column is washed in order to improve the purity of the eluted DNA. The final step is the elution of DNA from the column.

The third method (section 2.4), is based on cell wall disruption with only boiling and ultrasounds. Although being very rapid and promising in contaminated water samples, it is still not competent for food samples due to the high amount of inhibitors present.

The fourth method (section 2.4) is an effective phase separation technique using ultrasounds and boiling-based cell wall disruption and chloroform-isoamyl alcohol, in order to disrupt cellular integrity stabilizing DNA in the lysate and extract it with organic reagents into the aqueous phase. Published data has also referred and compared DNA extraction methods in the past [24]. According to Table 2, the fourth presented DNA extraction protocol was proved a potentially powerful tool for the isolation of bacterial DNA that provides great results in combination with almost the samerapidity and simplicity with the commercial kits.

On the other hand, this study dictates that LAMP technique has in most cases 10-fold greater sensitivity than conventional PCR for the detection and identification of pathogens. Published data also agree with the above statement [23],[25]. The results of this study's experiments, also agree with previously published results that compare the detection limits between LAMP and PCR [23],[25]. This report is probably justified because LAMP technique functions with the use of four to six primers designed to specifically amplify the target gene, giving higher specificity and sensitivity than the conventional PCR which functions with the use of two primers. Furthermore, the *Bst* polymerase used in LAMP, has very high activity, thus great amounts of DNA can be produced in short time [23],[26]. Moreover, another possible explanation is that the DNA extraction was conducted in food samples which are considered complexed matrices while containing many inhibitors. However, LAMP technique is proved more tolerant to inhibitors than PCR [15] and thus is probable to give better results in the detection of foodborne pathogens.

The general objective of this study was the development of an innovative routine method that will directly process complex matrices for fast and inexpensive screenings for the identification of pathogenic bacteria with minimal instrument requirements. Therefore, after evaluating all the tested DNA extraction methods and molecular techniques used for the detection and identification of the selected foodborne pathogens (*S*. Typhimurium, *L. monocytogenes*), the procedure was carried out in commercial food samples.

Noting that the pathogen detection and identification of method 4 (section 2.4) in conjunction with the LAMP technique gave exactly the same results in 60 commercial food samples with PCR-electrophoresis (a gold standard method) after DNA extraction using two commercial kits, proves that the technique is very sensitive and specific. All the experiments gave also the same results after tested by a reference microbiological conventional culture (as described at section 2.7). The abovementioned, dictates that the fourth DNA extraction protocol in conjunction with the LAMP molecular technique could provide a cost-effective test for the identification of the most common causative bacteria to provide early identification and thus reduce the contaminations of food products. This specific protocol could-in the future- be legally validated and reach an important position in decision making (e.g., food recalls) for the food industry.

## 5. Conclusions

Even though food production and technology are growing rapidly, food safety doesn't seem to keep up accordingly.

The present study reports a comparison between an in-house method for the DNA extraction of foodborne pathogens in contaminated food samples, based on the chloroform-isoamyl alcohol protocol, with DNA extraction using only boiling and ultrasound processing of the samples and DNA extraction using two commercial kits (Qiagen, Macherey-Nagel).

This particular almost instrument-free DNA extraction protocol (since it requires only centrifuge, freezer, heating block and ultrasonic bath) presents significantly similar limits of detection, compared to the other commercial kits for DNA extraction, while requires almost the same amount of time with much lower costs, for each processed sample. Moreover, it is statistically as sensitive to both gram negative and gram positive bacteria. Therefore, it might be rendered a very promising technique that could replace other commercial methods for DNA extraction in the near future.

Regarding the molecular techniques used to identify bacterial DNA by *S*. Typhimurium and *L. monocytogenes*, both PCR and LAMP showed similar sensitivities, with the later showing a slightly lower LOD in certain results and much more rapidity. However, in food safety monitoring procedures, even small differences in the methods' LODs could be considered critical. LAMP technique also, does not need expensive instrumentation, such as a thermal cycler, in order to operate due to its isothermal conditions, but can be carried out in a thermal water bath or even a conventional oven. As a result of this study, the use of the chloroform-isoamyl alcohol protocol based DNA extraction method, in combination with the LAMP technique, was proved to be as sensitive as the identification using commercial kits in combination with PCR–electrophoresis. Still, the first procedure can be conducted in large scales much more rapidly, simply and without the need of expensive and complexed instrumentation, giving the companies a great opportunity to monitor their food production in the future.
